# A Psychological Problem. (?)

**Published:** 1851-04-01

**Authors:** 


### A PSYCHOLOGICAL PROBLEM. (?)

CHARLES HENRY ACHERLY, a retired lieutenant in the navy, was recently tried at Swansea for manslaughter. The prisoner is well known in London, as "Captain Acherly," for his attendance at public meetings, and demonstrations of his "triangular theory," in the public parks of the metropolis. He is a man of position—a magistrate for Worcestershire, and of education—but an enthusiast. He stood indicted for causing, by quack treatment, the death of Matthew Tingle, a collier, who had been scorched in a mining explosion at Aberdare. On the 12th December, the prisoner came to the house of Tingle, and stated that he was a "doctor from Lancashire," who had orders from the owner of the mine to treat the suffering collier medically. Tingle was then in the dangerous stage which follows extensive scorching. The accident had removed the skin off from a large portion of the body. The regular doctor of the mining-works considered that he "had a chance." The prisoner ordered all the plasters to be removed, so as to expose the raw surface to the air; he placed a lamp of peculiar construction under the man's nose, to make him breathe hot air, and had his limbs smartly agitated by attendants. The collier died in a few hours afterwards; and Dr. Davies swore that this treatment, especially the exposure of the wounds and the agitation of the limbs, *accelerated* the death, though he could not be sure that the patient would otherwise have lived. The counsel for the crown admitted that the prisoner's motive was good; but urged that, by acting with "ignorance, or gross negligence, or misconduct," he had, through accelerating the death, been guilty of manslaughter. The prisoner conducted his own defence: at his side was a lamp of his own "invention," and before him was a pile of antique folios and quartos. He spoke with fluency, and in an effective manner, for an hour and a half; and grounded his defence on the most ludicrous mixture of the exploded physical dogmata of the alchemists and schoolmen of the ante-Baconian times, with misinterpreted modern science and Biblical lore. He started from the time-worn axiom, that "Nature abhors a vacuum;" the amusing anatomical specialty that there is "a circulation of air between the periosteum and the bones;" and the droll assumption that, by means of his lamp, he can "remove the atmospheric tonnage" from the human surface. These points he worked up with quotations from his old authors, and from the Bible, and with some remarks of really striking sense, into an extraordinary web of defence. At the time when the colleges were removed from Cricklade to Oxford [by Alfred the Great], there was "a putrefaction in literature;" and even now "the treatment of disease goes on rules laid down prior to the Reformation." In Aristotle's first book—"On Vacuity"—the Stagyrite says, "there is voidness;" on that foundation the prisoner would stand or fall. Holding up a feather, he referred to the common physiological tenet that some birds have the power of lessening their specific gravity by exhausting the quill part of their feathers of the air in them; and then he declared that, by putting the feather down Tingle's throat, he contributed to lighten the circulation of air between the periosteum and his bones. The Bible says, in Psalm cii. verse 5, "By reason of my groaning"—"that (the groaning) is the same thing with the rattles in Matthew Tingle's throat." Towards the end of his extraordinary effusion, the prisoner referred with tact to subjects which would influence a Welsh jury,—his opposition to the introduction of Rural Police; his magistracy; his temperate life—the life of an inventive genius; and, above all, to his descent from Prince Llewellyn and Owen Tudor. The jury gave an hour's deliberation to their verdict, and then found the prisoner "Not guilty;" whereupon the spectators in court shouted applause.

